# Co-administration of avian influenza virus H5 plasmid DNA with chicken IL-15 and IL-18 enhanced chickens immune responses

**DOI:** 10.1186/1746-6148-8-132

**Published:** 2012-08-06

**Authors:** Kian-Lam Lim, Seyed Davoud Jazayeri, Swee Keong Yeap, Noorjahan Banu Mohamed Alitheen, Mohd Hair Bejo, Aini Ideris, Abdul Rahman Omar

**Affiliations:** 1 Institute of Bioscience, University Putra Malaysia, Serdang, Selangor, 43400, Malaysia; 2 Faculty of Veterinary Medicine, University Putra Malaysia, Serdang, Selangor, 43400, Malaysia; 3 Faculty of Biotechnology and Biomolecular Sciences, University Putra Malaysia, Serdang, Selangor, 43400, Malaysia

**Keywords:** Avian influenza virus, DNA vaccine, H5, IL-15, IL-18, CD4+ T cells, CD8+ T cells

## Abstract

**Background:**

DNA vaccines offer several advantages over conventional vaccines in the development of effective vaccines against avian influenza virus (AIV). However, one of the limitations of the DNA vaccine in poultry is that it induces poor immune responses. In this study, chicken interleukin (IL) -15 and IL-18 were used as genetic adjuvants to improve the immune responses induced from the H5 DNA vaccination in chickens. The immunogenicity of the recombinant plasmid DNA was analyzed based on the antibody production, T cell responses and cytokine production, following inoculation in 1-day-old (Trial 1) and 14-day-old (Trial 2) specific-pathogen-free chickens. Hence, the purpose of the present study was to explore the role of chicken IL-15 and IL-18 as adjuvants following the vaccination of chickens with the H5 DNA vaccine.

**Results:**

The overall HI antibody titer in chickens immunized with pDis/H5 + pDis/IL-15 was higher compared to chickens immunized with pDis/H5 (p < 0.05). The findings revealed that the inoculation of the 14-day-old chickens exhibited a shorter time to achieve the highest HI titer in comparison to the inoculation of the 1-day-old chickens. The cellular immunity was assessed by the flow cytometry analysis to enumerate CD4+ and CD8 + T cells in the peripheral blood. The chickens inoculated with pDis/H5 + pDis/IL-15 demonstrated the highest increase in CD4+ T cells population relative to the control chickens. However, this study revealed that pDis/H5 + pDis/IL-15 was not significant (P > 0.05) in inducing CD8+ T cells. Meanwhile, with the exception of Trial 1, the flow cytometry results for Trial 2 demonstrated that the pDis/H5 + pDis/IL-18 inoculated group was able to trigger a higher increase in CD4+ T cells than the pDis/H5 group (P < 0.05). On the other hand, the pDis/H5 + pDis/IL-18 group was not significant (P > 0.05) in modulating CD8+ T cells population in both trials. The pDis/H5 + pDis/IL-15 inoculated group showed the highest IL-15 gene expression in both trials compared to other inoculated groups (P < 0.05). Similar results were obtained for the IL-18 expression where the pDis/H5 + pDis/IL-18 groups in both trials (Table 8) were significantly higher compared to the control group (P < 0.05). However, the expressions of other cytokines remained low or undetected by GeXP assay.

**Conclusions:**

This study shows the diverse immunogenicity of pDis/H5 co-administered with chicken IL-15 and IL-18,with pDis/H5 + pDis/IL-15 being a better vaccine candidate compared to other groups.

## Background

Avian influenza (AI) is regarded as one of the most important diseases infecting poultry and other birds throughout the world. Outbreaks of H5N1, the highly pathogenic avian influenza (HPAI), in poultry all over Asia have caused significant economic and public health difficulties [1]. Traditionally, the control of the HPAI has been achieved through the culling of infected flocks. However, the culling of infected poultry flocks alone has not proven to be sufficient in the eradication of the disease as the HPAI is endemic to a large geographical area, with the virus infecting poultry and indigenous wildfowl [2]. In 2005, in accordance with the recommendations of the OIE/FAO in collaboration with the WHO, the vaccination of uninfected flocks was deemed as one of the measures to prevent the outbreak and spread of the disease [2]. In addition, the immunization of poultry can limit the source of the virus that is responsible for its transmission to human or mammalian hosts [3]. Therefore, the development of an efficacious vaccine against the avian influenza virus (AIV) is desirable.

The main advantages of DNA immunization approaches include simplicity of delivery, inexpensive rapid mass production, and ease of preparation [4] without the safety concern of incomplete inactivation in the inactivated whole virus vaccine, which may cause outbreaks [5]. Moreover, DNA vaccines were also able to induce both humoral and cell-mediated immune responses [6]. Despite the advantages mentioned, low immunogenicity and expression levels, low transfection efficiency and targeting specificity remain the main obstacles compared to conventional vaccines [6,7]. Thus, the employment of adjuvants might help to overcome the above obstacles and enhance the vaccine-induced immune responses.

Genetic adjuvants are expression vectors encoding biologically active molecules such as cytokines, chemokines, soluble forms of cell ligands, adhesion proteins, or other immuno-reactive molecules [8]. Although avian cytokines have been poorly defined, they have the potential to control infectious diseases and can act as vaccine adjuvants that may specifically activate immune systems leading to effective protection [9]. It has been shown that IL-15 in mammals has a major role in natural killer cell development and proliferation, T cell growth, B cell proliferation and differentiation, and has an important influence on the development of CD4+ T cells [10,11]. In addition, IL-15 and IL-18 also play an important role in the development of T helper 1 cells [12] and were able to yield stronger CD4+ T cells, CD8+ T cell responses [13] and induction of NK cells [14]. Hence, the purpose of the present study was to explore the role of chicken IL-15 and IL-18 as adjuvants following the vaccination of chickens with the H5 DNA vaccine.

## Methods

### Construction of recombinant plasmids DNA

The pDis/H5 plasmid was constructed by sub-cloning the HA gene of AIV A/Chicken/Malaysia/5744/04 (H5N1) from pCR2.1/H5 into the plasmid expression vector pDisplay (Invitrogen, USA). The chicken IL-15 [GenBank: AY005476] and IL-18 [GenBank: AJ277865] clones were obtained from the Delaware Biotechnology Institute, Newark DE, USA. The H5, IL-15 and IL-18 genes were amplified using forward and reverse primers with *Sac*II and *Acc*I restriction sites respectively (Table 1), and ligated to the pDisplay vector. The selected recombinant clones were further confirmed by restriction enzyme analysis and sequencing.

**Table 1:** List of primers used in amplification and cloning of AIV genes

**Primer name**	**Primer sequence**	**Gene size (bp)**
H5f*Sac*II	5’ CCC CCG CGG ATG GAG AAA ATA GTG CTT 3’	H5 gene/1722
H5r*Acc*I	5’ CCC GTC GAC AAT GCA AAT TCT GCA 3’	
IL-15f*Sac*II	5’ AAA CCG CGG ATG CTG GGG ATG GCA CAG 3’	IL-15 gene/564
IL-15r*Acc*I	5’ GGG GTC GAC CTG GTA AGT TGC AAA TCT TGC 3’	
IL-18f*Sac*II	5’ CCC CCG CGG ATG AGC TGT GAA GAG ATC 3’	IL-18 gene/486
IL-18r*Acc*I	5’ CCC GTC GAC AAA GGC CAA GAA CAT TCC TTG 3’	

### Transfection

The Vero cells were seeded in a 6-well plate with a DMEM medium (Gibco, USA) without antibiotics one day before the transfection to achieve 90-95% confluence at the time of transfection. The transfection of the plasmid was performed using Lipofectamine (Invitrogen, USA). A mixture of 3 μg purified plasmid DNA and 6 μL of Lipofectamine (Invitrogen, USA) reagent was added to the monolayer cells. After a 48-hour post-transfection period, the cells were trypsinized and sub-cultured in new flasks with a DMEM medium containing Geneticin (Invitrogen, USA). The medium was replaced every 4 days for the next 14 days. The cells were harvested and used for further analysis.

### Western blotting

The transfected cells were washed with 1X PBS and mixed with an equal volume of a 2X sample buffer [0.5 M Tris (pH 6.8), 100% glycerol, 10% (w/v) SDS, 0.5% (w/v) bromophenol blue, 10% (v/v) β-mercaptoethanol] and short spun before being heated at 100°C for 10 minutes. The protein samples were run on 12% SDS-PAGE and thereafter subjected to electro-transfer to a nitrocellulose membrane with a constant voltage of 15 volts for 90 minutes. The membrane was incubated with a primary rabbit polyclonal antibody against the avian influenza H5 antibody (Abcam, USA) for 1 hour. The membrane was washed with washing solution (Invitrogen, USA) and the process was repeated thrice. The membrane was then incubated with Rabbit Secondary Antibody Solution (Invitrogen, USA) for 30 minutes and further washed thrice. Finally, the blot was incubated with 5 mL of Chromogenic Substrate solution (BCIP/NBT substrate for alkaline phosphatase) (Invitrogen, USA) until the bands appeared.

### Indirect immunofluorescence test

The expressed proteins were subjected to the immunofluorescence test using a polyclonal antibody raised in rabbits and a secondary fluorescein isothiocyanate (FITC) anti-rabbit antibody raised in goats (KPL, USA). The stable transfected Vero cells were seeded in a Lab-Tek™ chamber slide (Nunc, USA) and grown to 70% confluence. The confluent cells were rinsed with 1X PBS and overlaid with a primary rabbit polyclonal antibody against the avian influenza H5 antibody (Abcam, USA) diluted at 1:1000 and incubated at 37°C for 1 hour. The chamber slide was washed 3 times with 1X PBS and FITC anti-rabbit antibody (Abcam, USA) diluted at 1:500, added onto the cells and incubated at 37°C for 1 hour. This was followed by washing the slide 3 times with PBS and it was then air-dried and mounted in glycerol : 1X PBS (9:1). The mounted slide was then viewed under an inverted phase contrast microscope with fluorescent light (Olympus, USA).

### Reverse transcription-polymerase chain reaction

A RT-PCR analysis was performed on the Vero cells to determine the presence of pDis/H5, pDis/IL-15 and pDis/IL-18 mRNA transcripts. Briefly, the total RNA was extracted using Trizol reagent (Invitrogen, USA) and treated with 2 mL of DNase I (Sigma, UK) at 37°C for 30 minutes and the reaction was stopped with 2 mL of 50 mM EDTA and heat inactivation at 56°C for 10 minutes. The extracted RNA was subjected to RT-PCR using the One Step Access RT-PCR System (Promega, USA). Amplification was performed in a 25 μL RT-PCR mixture containing the AMV/*Tfl* 1X Reaction Buffer, 0.2 mM of dNTP mixture, 3.0 mM MgSO_4_, 0.5 μM of each primer (Vivantis, Malaysia), 0.8 U RNasin Ribonuclease Inhibitor (Promega, USA), 0.1 U AMV Reverse Transcriptase (Promega, USA), 0.1 U*Tfl* DNA Polymerase (Promega, USA) and 10 μg of RNA. The reaction mixture was incubated at 45°C for 45 minutes. The amplification was performed as follows: initial denaturation at 95°C for 2 minutes followed by 30 cycles of denaturation at 95°C for 30 seconds, annealing at 55°C for 44 seconds and extension at 68°C for 2 minutes. Lastly, the mixture was subjected to a final extension at 68°C for 10 minutes.

### Immunization of chickens

The plasmids pDis/H5, pDis/IL-15 and pDis/IL-18 were extracted and purified using the EndoFree Plasmid Mega Kit (Qiagen, Germany) according to the manufacturer’s procedure. In the first trial, five groups of chickens were established with ten specific-pathogen-free (SPF) chickens per group. The groups were as follows: Group 1, control group without inoculation; Group 2, control group inoculated with pDisplay vector; Group 3, vaccinated with pDis/H5; Group 4, vaccinated with pDis/H5 + pDis/IL-15, and Group 5, inoculated with pDis/H5 + pDis/IL-18. One-day-old chickens were injected twice with 150 μg of purified plasmid DNAs using 1 mL tuberculin syringes attached with 25 G x 5/8 inch needles with 100 μL of the plasmid DNA solutions through an i.m route on the left pectoral muscle of the chickens. A booster dose was administered at 2 weeks post inoculation. Bleeding was performed via the jugular vein and the serum was collected from the 1-day-old chickens at 1, 2, 3, 5, 6 and 7 weeks post inoculation. Blood lymphocytes were collected from the 1-day-old chickens at 1 and 5 weeks post inoculation. The chickens in each group were euthanized at 7 weeks post inoculation. In the second trial, five groups of chickens were established with seven SPF chickens per group. Fourteen-day-old chickens were injected twice with 150 μg of purified plasmid DNAs as described in Trial 1. A booster dose was administered at 3 weeks post inoculation. Bleeding was performed via the jugular vein and the serum was collected from the 14-day-old chickens at 3, 4 and 6 weeks post inoculation, whilst blood lymphocytes were collected from the 14-day-old chickens at 3 and 6 weeks post inoculation. The chickens in each group were euthanized at 6 weeks post inoculation. The experimental trials were approved by the Animal Care and Use Committee at the Faculty of Veterinary Medicine, Universiti Putra Malaysia, reference number, UPM/FPV/PS/3.2.1.551/AUP-R80.

### Hemagglutination inhibition (HI) test

The standard HI assay was done on a 96-well V-shape microtiter plate following the method described by Vogel and Shelokov [15]. A low pathogenic inactivated AIV H5N2, A/duck/Malaysia/8443/04 (H5N2) was obtained from the Veterinary Research Institute, Ipoh. The virus was titrated at four hemagglutinin activity units (HAU), 4 HA/25 μL. After incubation of the antigen for 15 minutes to allow for reaction with the serum, 25 μL of 0.5% (v/v) chicken RBCs was added to all the test wells and incubated for 20 minutes. The agglutination of the RBCs indicated the absence of antibodies against the AIV H5N1.

### Detection of the DNA vaccine distribution *in vivo*

A PCR analysis was performed on the spleen and muscles to determine the presence of the pDis/H5, pDis/IL-15 and pDis/IL-18 genomic DNA. The genomic DNA was extracted from the tissues using 10% sodium dodecyl sulphate (SDS) and 20 mg/mL of Proteinase K (Invitrogen, USA). The lysate was extracted by phenol chloroform isoamyl alcohol (25:24:1) and chloroform isoamyl alcohol (49:1). The precipitated DNA was used as PCR templates for detecting the distribution of DNA vaccines *in vivo*. A RT-PCR analysis was performed on the spleen and muscles to determine the presence of the pDis/H5, pDis/IL-15 and pDis/IL-18 mRNA transcripts based on the methods described in previous section. The RNA preparations were standardized by the RT-PCR for β-Actin and were free from DNA contamination evaluated by the lack of amplification following non-reverse transcribed RNA using the same samples and set of primers (Table 2).

**Table 2:** List of primers used in amplification of mRNA transcript of AIV and chicken cytokine genes

**Primer name**	**Primer sequence**	**Gene size (bp)**
H5f	5’ TGG GAG GAT GGA GTT CTT CTG GAC 3’	H5 gene/175
H5r	5’ TGG AGT TTG ACA CTT GGT GTT GCA 3’	
IL-15f	5’ AAC TTG CTC CAT AGG TTT CCG A 3’	IL-15 gene/126
IL-15r	5’ AGA GTT TTG TGT TGG CTG TGC CAT 3’	
IL-18f	5’ TTC GAA ACG TCA ATA GCC AGT TGC 3’	IL-18 gene/161
IL18r	5’ GGG GTC GAC ATT GTC ATA TTC CTC TGC 3’	
ß-Actinf	5’ CAT CTA TGA AGG CTA CGC CCT 3’	ACTB gene/155
ß-Actinr	5’ GCT TCT CCT TGA TGT CAC GCA CAA 3’	

### Analysis of CD3 + /CD4+ and CD3 + /CD8+ T-lymphocytes

Chicken peripheral blood samples were collected from the jugular vein in 2.5 ml syringes containing 0.2 ml of sodium heparin to prevent clotting. The peripheral blood mononuclear cells (PBMC) were isolated from the heparinized chicken’s blood, as described in Ficoll-Paque PLUS protocol, and a single cell suspension was prepared at a concentration of 1 x 10^7^ cells/mL. Then, 100 μL of cell suspensions (1 × 10^6^ cells) were incubated for 2 hours at 4°C. The chicken lymphocytes were double-stained with mouse anti-chicken CD3-R-phycoerythrin (R-PE) (Southern Biotech, USA) and mouse anti-chicken CD4-FITC (Southern Biotech, USA) or mouse anti-chicken CD3-R-phycoerythrin (R-PE) (Southern Biotech, USA) and mouse anti-chicken CD8α-FITC (Southern Biotech, USA). The immuno-stained cells were analyzed on a FACSCalibur flow cytometer (Becton Dickinson, San Jose, CA, USA). The small lymphocyte population was differentiated according to cell size as defined by the forward scatter (FSC) value and their granularity as defined by the side scatter (SSC) value using the FSC/SSC dot plot diagram and subjected to four quadrant statistics for differentiation of single T cell sub-populations.

### Cytokine gene expression analysis

The expression of selected cytokines from frozen spleen samples was performed using the GenomeLab GeXP (Beckman Coulter, USA). In brief, the RNA was extracted using Trizol reagent according to the manufacturer's instructions (Invitrogen, USA). A total of 13 cytokine genes, an internal control and a housekeeping gene (COL6A2) were designed using the GenomeLab eXpress Profiler software (Table 3). The reverse transcription reactions and PCR amplification were performed according to the GenomeLab GeXP Start Kit using the Beckman–Coulter protocol. The PCR product sizes were determined using the GenomeLab GeXP software and were compared to the expected PCR product size to identify each transcript. The data was imported into the analysis module of the eXpress Profiler software. Relative gene expressions of the individual genes were normalized by dividing the peak area of each gene by the peak area of the COL6A2 gene following the method described by Sadava *et al.*[16].

**Table 3:** Gene name, product size, and forward and reverse primer sequences of GeXP assays of chicken spleen

**Gene name**	**Fragment size**	**Left sequence without universals**^**c**^	**Right sequence without universals**^**d**^
IL-15	187	AAGGTTTCCGAGGCTTGTACC	GTTTTCTGACTCTCCGGCCT
IL-12β	194	TGAAGGAGTTCCCAGATGCT	AAGAACGTCTTGCTTGGCTC
IL-2	201	TAGTCACGAGGGGACCTTTG	GACGGCTTTCTTTGATGAGC
IL-1β	208	TCTTTGGCTGTATTTCGGTAGC	TGCACTCCTGGGTCTCAGTT
IL-18	215	CAAAGTGTACACCATCCTGGAA	AAAGACGATTCCGCTTTCTTC
IL-4	222	CAAGTCAAAGCCGCACATC	GAGGATCCACCAGCTTCTGT
IL-6	236	TCAACATCGCCACCTACAAG	TAAAAGCAACGGGACGGTAA
IL-10	243	GCTTGTGGTTCGTCCAGATT	GAACAACCATTTTCCCATGC
IL-8	250	GAGCATCCGGATAGTGAATGA	TTGAGGGAGGTGCTGCTG
IFN-γ	257	CAACGCAGAAGAAACAGTGC	CCCCATTGCAAATGTTGTATC
TNFSF13B	264	AGCCACAGCATCTTCTTCGT	TCGAAGGAGAGCCACTCATC
TGFβ	271	CAACCCCCAATGTCTCTGTT	ACCAGGAAACAAGCTTGACG
GMCSF	279	CCGTTTCAGGAACCAGAGAG	GTCTGGCTGCTGGACATTTT
COL6A2^a^	287	ATTTTCAATCCAGGGACGAT	CTCCTCTTCTCGCAGGTGAA
KAN^b^	325	ATCATCAGCATTGCATTCGATTCCTGTTTG	ATTCCGACTCGTCCAACATC

^a^ Gene used for normalization.

^b^ Internal control.

^c^ Forward universal primer sequence (AGGTGACACTATAGAATA).

^d^ Reverse universal primer sequence (GTACGACTCACTATAGGGA).

### Statistical analysis

An ANOVA was performed using the SPSS Windows program version 12.0 to analyse the differences between animal immunization groups under each of the above three indices (the percentage of CD3 + /CD4+ and CD3 + /CD8+ subgroups of peripheral blood T-lymphocytes, the antibody responses and the GeXP multiplex gene expression were compared with those of the control animals). A P-value of less than 0.05 was considered as significant.

## Results

### Construction of DNA vaccines

**Figure 1:**
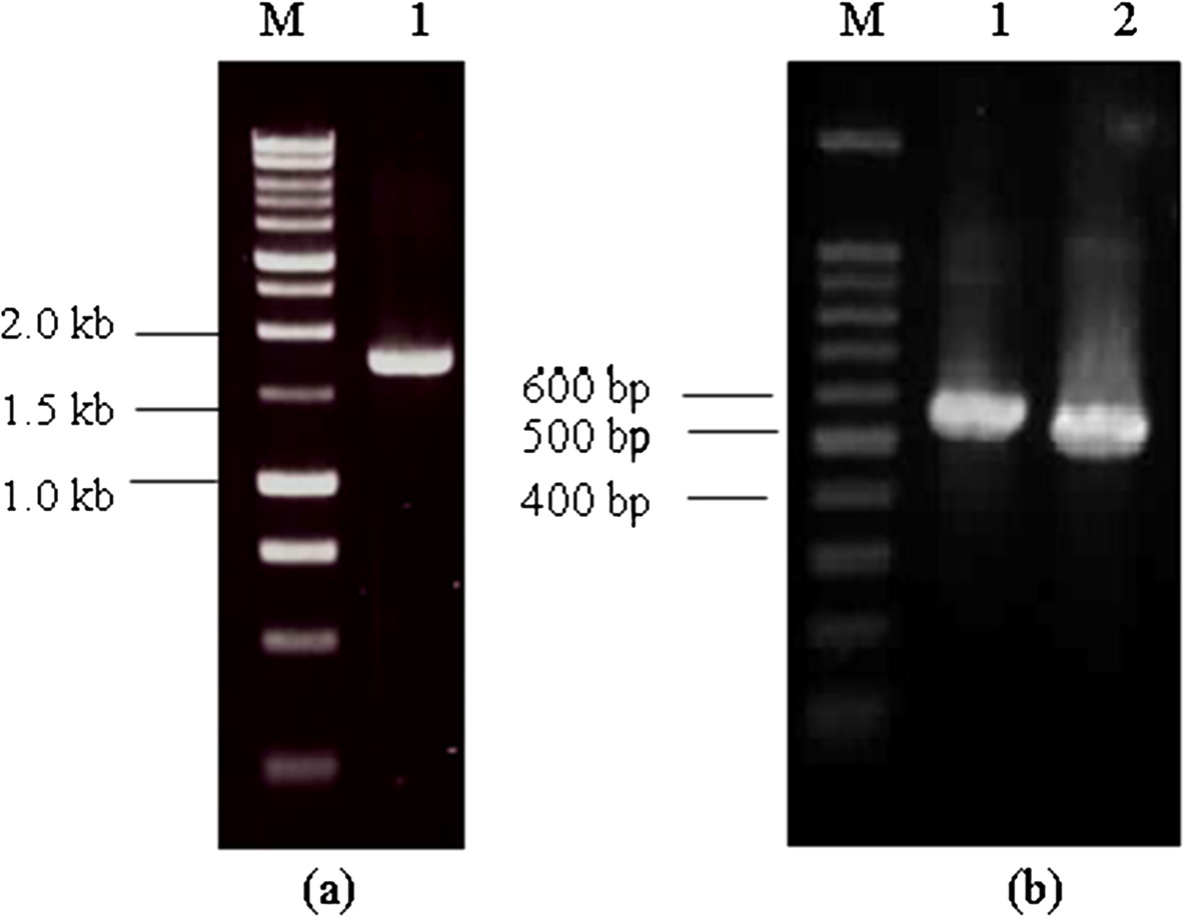
**PCR amplification of (a) H5 gene from pCR2.1 vector and (b) IL-15 and IL-18 genes from pgm2n.pk009.p17 and pgm2n.pk010.c11, respectively.** The DNA band was observed in (**a**) 1% (w/v) agarose gel. M: 1 kb DNA ladder (Promega, Madison WI); Lane 1: H5 gene (1722 bp) and (**b**) 2% (w/v) agarose gel. M: 100 bp DNA ladder (Promega, Madison, WI). Lane 1: IL-15 gene (564 bp); Lane 2: IL-18 gene (486 bp).

The H5, IL-15 and IL-18 genes were amplified by PCR and cloned into purified digested pDisplay vectors with designations of pDis/H5, pDis/IL-15 and pDis/IL-18, respectively. The resulting H5, IL-15 and IL-18 PCR products appeared in agarose gel as DNA bands at expected sizes of 1722 bp, 564 bp and 486 bp, respectively (Figure 1).

### Expression of the recombinant plasmids in Vero cells

**Figure 2:**
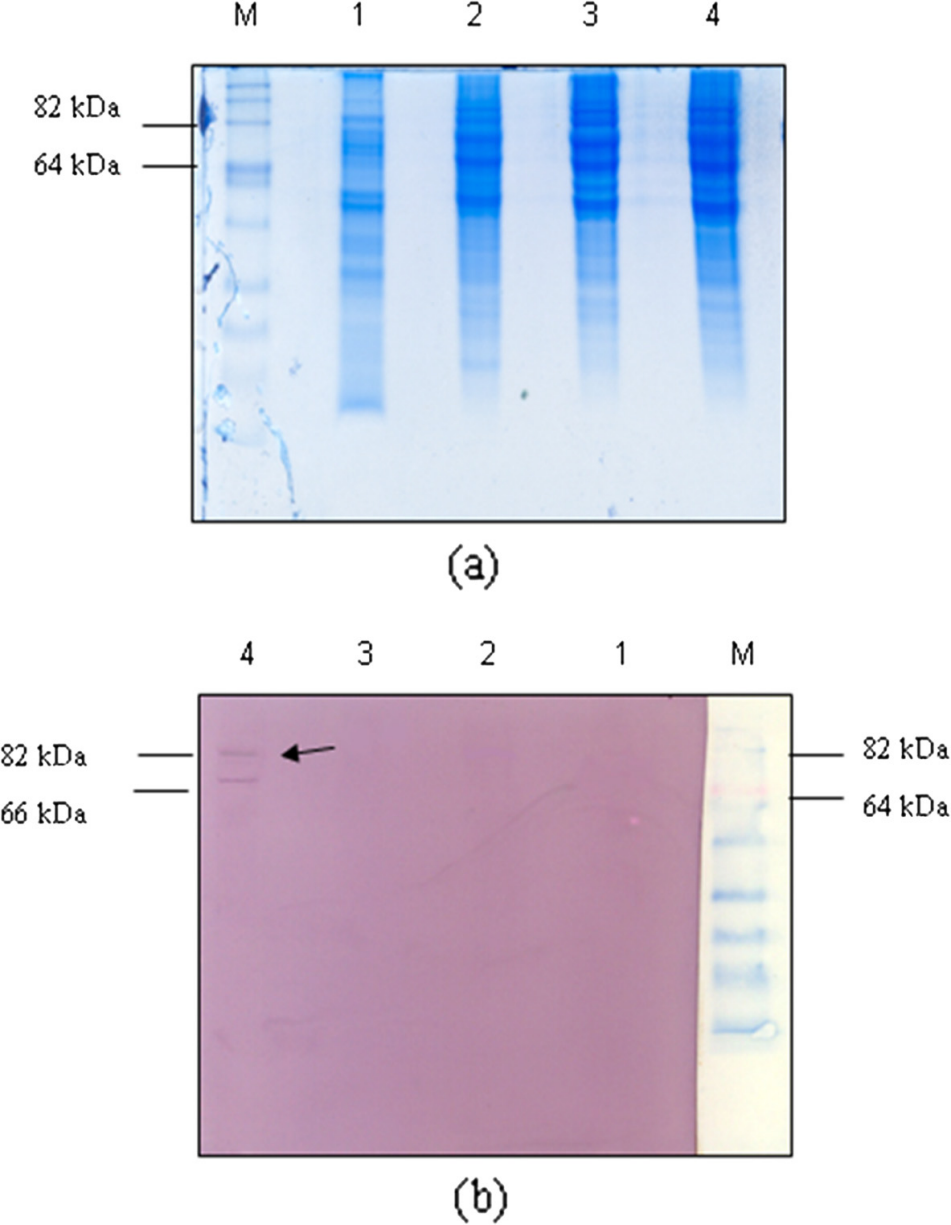
**SDS-PAGE and Western blot analysis of Vero cells transfected with recombinant plasmid, pDis/H5.** (**a**) Cell lysates were separated on 12% SDS-PAGE (**b**) Western blot with transferred protein samples incubated with primary antibodies against H5 of AIV and anti-rabbit secondary antibodies conjugated with alkaline phosphatase. M: BenchMark^TM^Pre-Stained Protein Ladder. Lane 1: Non-transfected Vero cell lysates; Lane 2: pDisplay vector; Lane 3: pDis/H5 vector 1; and 4: pDis/H5 vector 2. Distinct band of 82 kD which corresponds to the H5 protein of AIV was detected in lane 4 (arrowhead) whilst, pDis/H5 vector 1 construct failed to show H5 expression.

**Figure 3:**
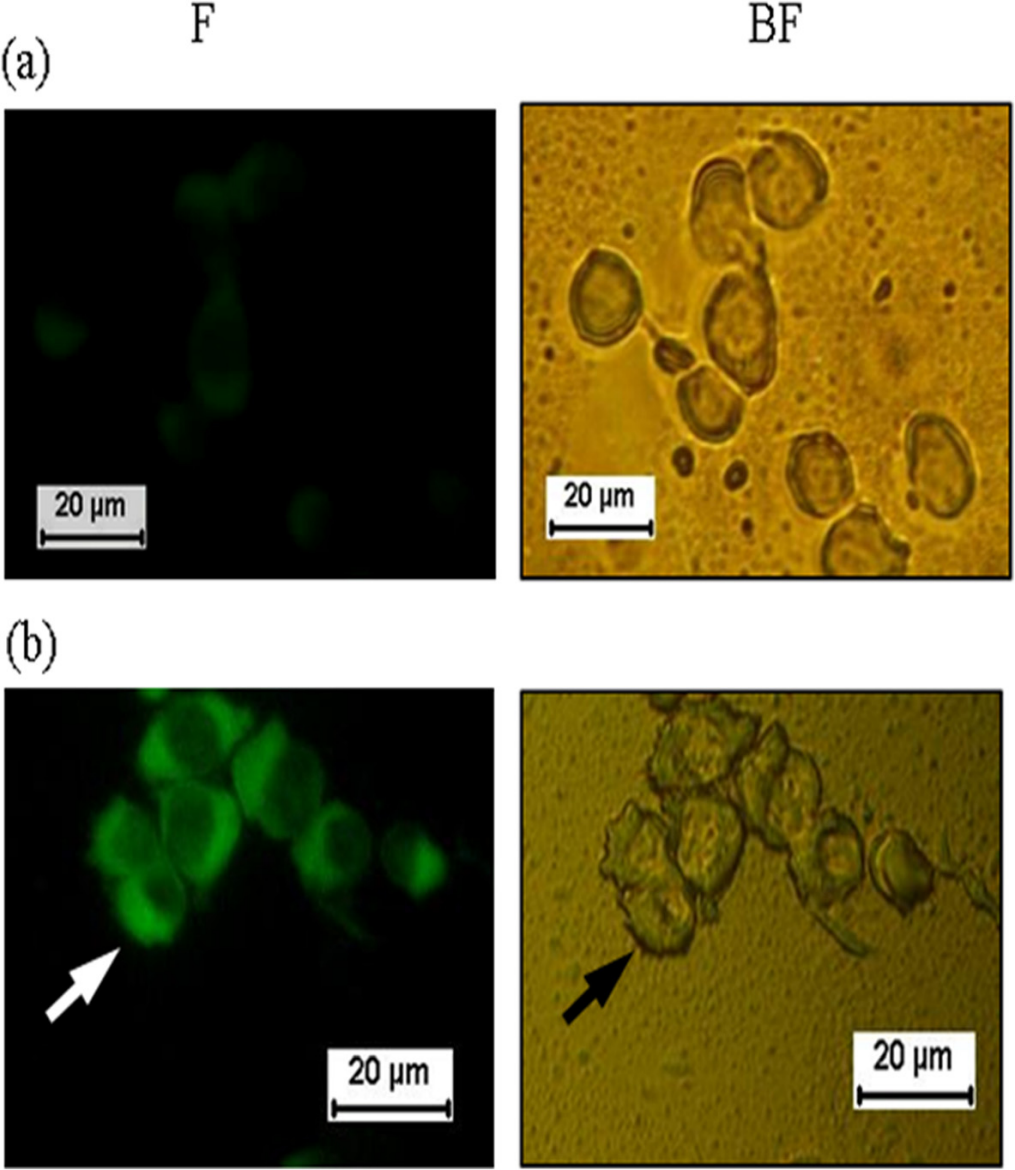
**Stable expression of Vero cells with (a) pDisplay vector, (b) pDis/H5.** Recombinant H5 proteins expression was detected by IFAT assay and visualized in bright field (BF) and fluorescence (F). Vero cells transfected with pDisplay vector as negative control.

The Western blot analysis, using rabbit anti-H5 polyclonal antibody, was reacted with the recombinant proteins at 2 weeks post-transfection. Two different constructs of the H5 gene were analyzed for their ability to express protein in Vero cells. The recombinant H5 protein appeared as a band with a molecular weight of approximately 82 kDa (lane 4, Figure 2b). The observation of multiple bands in pDis/H5 (66 kDa) might be due to nonspecific binding by the polyclonal antibody used in the studies. However, an analysis of another construct showed no expression of the H5 protein (lane 3, Figure 2b). This might be due to the inability of the construct to express the H5 protein. An indirect immunofluorescence antibody test (IFAT) was performed to further confirm the expression of the recombinant protein. As shown in Figure 3, the cells transfected with pDis/H5 exhibited a bright green fluorescence showing that individual constructs encoding the H5 protein can be expressed in the eukaryotic system. No fluorescent staining was detected from cells that were transfected with the pDisplay vector only.

### Detection of mRNA transcript in Vero cells

**Figure 4:**
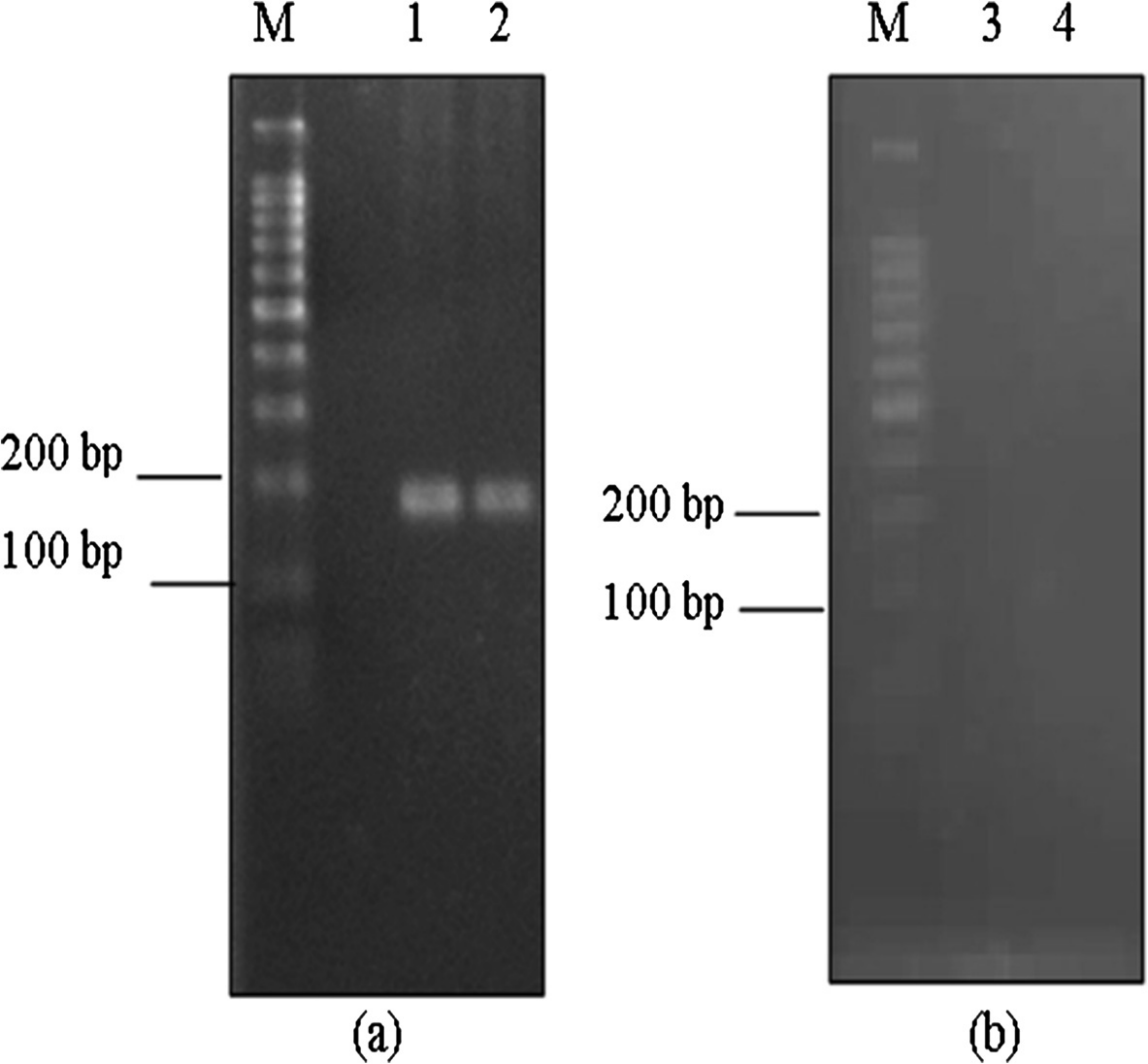
**RT-PCR analysis of total RNA extracted from transfected Vero cells.** RT-PCR analysis of the transfected Vero cells showed the presence of H5 transcript in the transfected cell lysate. The products were resolved in 2% (w/v) agarose gel in TAE. M: 100 bp DNA ladder (Promega, Madison WI); Lane 1 and 2: H5 transcript (175 bp) (**b**) No specific band was observed when RNAs was analyzed by PCR without the RT part as in Lane 3 and 4: H5.

**Figure 5:**
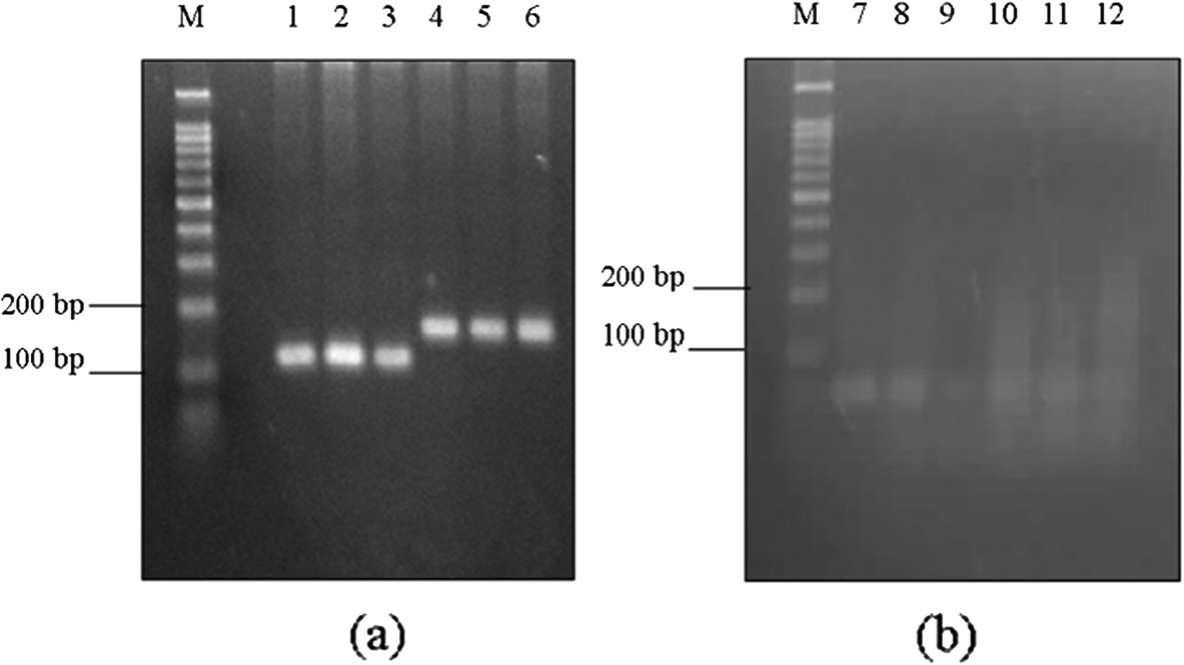
**RT-PCR analysis of total RNA extracted from transfected Vero cells.** RT-PCR analysis of the transfected Vero cells showed the presence of IL-15 and IL-18 transcripts in the transfected cell lysate. The products were resolved in 2% (w/v) agarose gel in TAE. M: 100 bp DNA ladder (Promega, Madison WI); (**a**) Lane 1 to 3: IL-15 transcript (126 bp), Lane 4 to 6: IL-18 transcript (161 bp). (**b**) No specific band was observed when RNAs were analyzed by PCR without the RT part as in Lane 7 to 9: IL-15 and Lane 10 to 12: IL-18.

The RT-PCR was performed to confirm the presence of the H5, IL-15 and IL-18 mRNA transcripts in Vero cells. The RT-PCR analysis revealed that the recombinant plasmids were transcriptionally active in Vero cells based on the detection of transcripts of the expected size as in Figure 4 and Figure 5. In order to ensure that the detection of the band was associated with the H5, IL-15 and IL-18 transcripts and was not an amplification from the plasmid DNA, the extracted RNA samples were processed for PCR amplification without the reverse transcription part. No specific band was detected from the samples indicating that the amplification result was not from the plasmid DNA.

### HI Assay in SPF chickens inoculated with DNA vaccines

Injections of various DNA vaccine formulations yielded variable HI responses in Trial 1 (Table 4) and Trial 2 (Table 5). Serum samples were collected and the HI titer was not detected in all the chickens prior to the inoculation of the plasmid DNA. The HI titer was not detected in the control chickens inoculated with the expression vector without the insert, pDisplay, as in negative control. Chickens inoculated with the pDis/H5, pDis/H5 + pDis/IL-15 and pDis/H5 + pDis/IL-18 groups were able to develop the HI antibody titer in both trials respectively.

**Table 4:** HI titer of serum samples obtained from chickens inoculated with various plasmid DNAs in Trial 1

**DNAs- inoculated groups**			**HI titer**				
	**Weeks post inoculation**						
	**1**	2*	**3**	**4**	**5**	**6**	**7**
Control	0	0	0	0	0	0	0
pDisplay	0	0	0	0	0	0	0
pDis/H5	1.52 ±1.38	1.62 ±1.43	1.87 ±1.43	2.30 ±1.34	3.25 ±1.60	4.92 ±1.60	6.50 ±1.40
pDis/H5 + pDis/IL-15	1.52 ±1.43	1.62 ±1.60	5.28 ±1.43^a c^	6.50 ±1.40^a^	13.00 ±1.40^a c^	10.56 ±1.43^a c^	12.13 ±1.43^a c^
pDis/H5 + pDis/IL-18	1.52 ±1.43	2.30 ±1.55	3.25 ±1.40^b^	4.92 ±1.40^b^	6.50 ±1.40^b^	6.50 ±1.40	6.50 ±1.40

* Chickens were given a booster 2 weeks after initial inoculation. Statistically significant differences (P < 0.05) are indicated by a, pDis/H5 compared with pDis/H5 + pDis/IL-15; b, pDis/H5 compared with pDis/H5 + pDis/IL-18; c, pDis/H5 + pDis/IL-15 compared with pDis/H5 + pDis/IL-18.

**Table 5:** HI titer of serum samples obtained from chickens inoculated with various plasmid DNAs in Trial 2

**DNAs-inoculated groups**	**HI GMT**		
	**Weeks post inoculation**		
	3*	**4**	**6**
Control	0	0	0
pDisplay	0	0	0
pDis/H5	2.69 ± 1.45	3.28 ± 1.40	3.28 ± 1.40
pDis/H5 + pDis/IL-15	3.62 ± 1.30^c^	16.00 ± 0.00^a c^	11.89 ± 1.45^a c^
pDis/H5 + pDis/IL-18	2.21 ± 1.30	6.56 ± 1.95^b^	4.42 ± 1.87
			

* Chickens were given a booster 3 weeks after initial inoculation. Statistically significant differences (P < 0.05) are indicated by a, pDis/H5 compared with pDis/H5 + pDis/IL-15; b, pDis/H5 compared with pDis/H5 + pDis/IL-18; c, pDis/H5 + pDis/IL-15 compared with pDis/H5 + pDis/IL-18.

In Trial 1, three weeks after the booster inoculation, significant HI Geometric mean titer (GMT) was detected for the pDis/H5 + pDis/IL-15 group which increased to 13.00 ± 1.40 HI GMT (p < 0.05). In Trial 2, one week after the booster inoculation, significant HI titers were detected for the pDis/H5 + pDis/IL-15 group which increased to 16.00 ± 0.00 HI GMT (p < 0.05). The results indicated that the booster inoculation increased the sero conversion rate of the SPF chickens. Besides that, the results also indicated that the SPF chickens in Trial 2 exhibited a shorter time to achieve the highest HI titer which was 1 week after the booster inoculation compared to 3 weeks after the booster inoculation in Trial 1 for pDis/H5 + pDis/IL-15.

There was a significant increase of the HI titer induced by the pDis/H5 + pDis/IL-18 inoculation at 1 week to 3 weeks after the booster inoculation compared with the pDis/H5 group in Trial 1 (P < 0.05) (Table 4). However, the pDis/H5 + pDis/IL-18 group reached a plateau at 6.50 ± 1.40 HI GMT from 3 weeks to 5 weeks after the booster inoculation. The HI titer in other time points induced by chicken IL-18 was not statistically significant (P > 0.05) (Table 4). In Trial 2, the HI titers induced by the pDis/H5 + pDis/IL-18 at all the inoculation time points were not significant compared with the pDis/H5 group (P > 0.05) except at 4 weeks post inoculation (Table 5). In summary, the data showed that the pDis/IL-15 plasmid DNA was able to enhance the antibody production together with the pDis/H5 construct through the i.m. route of inoculation.

### CD3+/CD4+ and CD3+/CD8+ T cells population following pDis/H5, pDis/H5 + pDis/IL-15 and pDis/H5 + pDis/IL-18 inoculation

Prior to the inoculation of the plasmid DNA, the percentage of CD3+/CD4+ and CD3+/CD8+ T cells population were 2.92 ± 0.40 and 0.52 ± 0.24 in Trial 1 respectively. Meanwhile, the percentage of CD3+/CD4+ and CD3+/CD8+ T cells population were 2.64 ± 0.40 and 0.69 ± 0.24 in Trial 2 respectively. The level of the CD3+/CD4+ T cells population in the pDis/H5, pDis/H5 + pDis/IL-15 and pDis/H5 + pDis/IL-18 groups in Trial 1 (Table 6) and Trial 2 (Table 7) were significantly higher compared to the control group (P < 0.05). The pDis/H5 + pDis/IL-15 inoculated group showed the highest CD3+/CD4+ T cells population at 2 weeks and 5 weeks post inoculation in Trial 1 and at 3 weeks and 6 weeks post inoculation in Trial 2 respectively, compared to the other inoculated groups (P < 0.05). The pDis/H5 + pDis/IL-18 inoculated group in Trial 2 showed a significant CD3+/CD4+ T cells level compared to the pDis/H5 group at 3 weeks and 6 weeks post inoculation (P < 0.05). However, the pDis/H5 + pDis/IL-18 inoculated group in Trial 1 was not significant enough to induce the CD3+/CD4+ T cells at 2 weeks and 5 weeks post inoculation compared to the pDis/H5 (P > 0.05).

**Table 6:** Immunophenotyping of CD3+/CD4+ and CD3+/CD8+ lymphocytes from chickens following inoculation with various plasmid constructs in Trial 1

**DNAs inoculated groups**	**Trial 1**			
	**Weeks post inoculation**			
	**CD3+/CD4+**		**CD3+/CD8+**	
	**Week 2**	**Week 5**	**Week 2**	**Week 5**
Control	2.23 ± 0.60	1.55 ± 0.26	0.91 ± 0.30	1.79 ± 0.58
pDisplay	2.55 ± 0.36	1.76 ± 0.14	1.16 ± 0.46	2.00 ± 0.35
pDis/H5	4.53 ± 0.33 ^*^	4.19 ± 0.52^* b^	3.16 ± 0.56^* a b^	2.97 ± 0.55^* a b^
pDis/H5 + pDis/IL-15	6.98 ± 0.73^* a c^	6.28 ± 0.60^* a c^	1.94 ± 0.46^*^	1.64 ± 0.44
pDis/H5 + pDis/IL-18	4.46 ± 0.34^*^	3.45 ± 0.26^*^	2.31 ± 0.84^*^	1.95 ± 0.52

Each value represents the mean percentages of T cell sub-populations ± SD. The percentage of CD3+/CD4+ and CD3+/CD8+ T cells before inoculation are recorded as 2.92 ± 0.40 and 0.52 ± 0.24, respectively. Statistically significant differences (P < 0.05) are indicated by an asterisk (*), treated compared with untreated groups; a, pDis/H5 compared with pDis/H5 + pDis/IL-15; b, pDis/H5 compared with pDis/H5 + pDis/IL-18; c, pDis/H5 + pDis/IL-15 compared with pDis/H5 + pDis/IL-18.

**Table 7:** Immunophenotyping of CD3+/CD4+ and CD3+/CD8+ lymphocytes from chickens following inoculation with various plasmid constructs in Trial 2

**DNAs inoculated groups**	**Trial 2**			
	**Weeks post inoculation**			
	**CD3+/CD4+**		**CD3+/CD8+**	
	**Week 3**	**Week 6**	**Week 3**	**Week 6**
Control	2.96 ± 0.52	7.25 ± 0.71	1.50 ± 0.28	3.58 ± 1.15
pDisplay	2.83 ± 0.34	7.42 ± 0.47	1.51 ± 0.66	3.28 ± 0.71
pDis/H5	6.56 ± 0.26^*^	8.86 ± 0.17^*^	2.52 ± 1.00	5.49 ± 1.15^* a^
pDis/H5 + pDis/IL-15	10.65 ± 0.59^* a c^	13.59 ± 1.15^* a c^	2.26 ± 0.38	3.53 ± 0.59
pDis/H5 + pDis/IL-18	8.42 ± 1.08^* b^	11.61 ± 0.61^* b^	2.12 ± 0.81	4.22 ± 1.21

Each value represents the mean percentages of T cell sub-populations ± SD. The percentage of CD3+/CD4+ and CD3+/CD8+ T cells before inoculation are recorded as 2.64 ± 0.40 and 0.69 ± 0.24, respectively. Statistically significant differences (P < 0.05) are indicated by an asterisk (*), treated compared with untreated groups; a, pDis/H5 compared with pDis/H5 + pDis/IL-15; b, pDis/H5 compared with pDis/H5 + pDis/IL-18; c, pDis/H5 + pDis/IL-15 compared with pDis/H5 + pDis/IL-18.

The level of the CD3+/CD8+ T cells population in the pDis/H5, pDis/H5 + pDis/IL-15 and pDis/H5 + pDis/IL-18 groups in Trial 1 at 2 weeks post inoculation were significantly higher compared to the control as shown in Table 8 (P < 0.05). However, only the pDis/H5 inoculated group in Trial 1 at 5 weeks post inoculation showed significant CD3+/CD8+ T cells level compared to the control group (P < 0.05). Similarly, only the pDis/H5 inoculated group in Trial 2 at 6 weeks post inoculation showed significant CD3+/CD8+ T cells level compared to the control group (P < 0.05). Overall, the pDis/H5 + pDis/IL-15 does not influence the CD3+/CD8+ T cells level in the PBMC except at 2 weeks post inoculation in Trial 1 (P < 0.05).

**Table 8:** Relative expression level of IL-15 and IL-18 genes in inoculated SPF chickens compared to control SPF chickens

**DNAs inoculated groups**	**Trial 1**		**Trial 2**	
	**IL-15**	**IL-18**	**IL-15**	**IL-18**
Control	0.62 ± 0.10	0.13 ± 0.03	0.41 ± 0.07	0.10 ± 0.02
pDisplay	0.41 ± 0.05	0.11 ± 0.03	0.51 ± 0.02	0.15 ± 0.00
pDis/H5	1.38 ± 0.11^* b^	0.14 ± 0.06	1.20 ± 0.13^* b^	0.17 ± 0.03
pDis/H5 + pDis/IL-15	3.69 ± 0.51^* a c^	0.19 ± 0.05	3.53 ± 0.33^* a^	0.18 ± 0.04
pDis/H5 + pDis/IL-18	0.74 ± 0.06	0.45 ± 0.02^* b c^	0.95 ± 0.12^*^	0.69 ± 0.13^* b c^

Note: Each value represents the mean ± SD. Statistically significant differences (P < 0.05) are indicated by an asterisk (*), treated compared with untreated groups; a, pDis/H5 compared with pDis/H5 + pDis/IL-15; b, pDis/H5 compared with pDis/H5 + pDis/IL-18; c, pDis/H5 + pDis/IL-15 compared with pDis/H5 + pDis/IL-18.

### Detection of transgene in vaccinated SPF chickens

**Figure 6:**
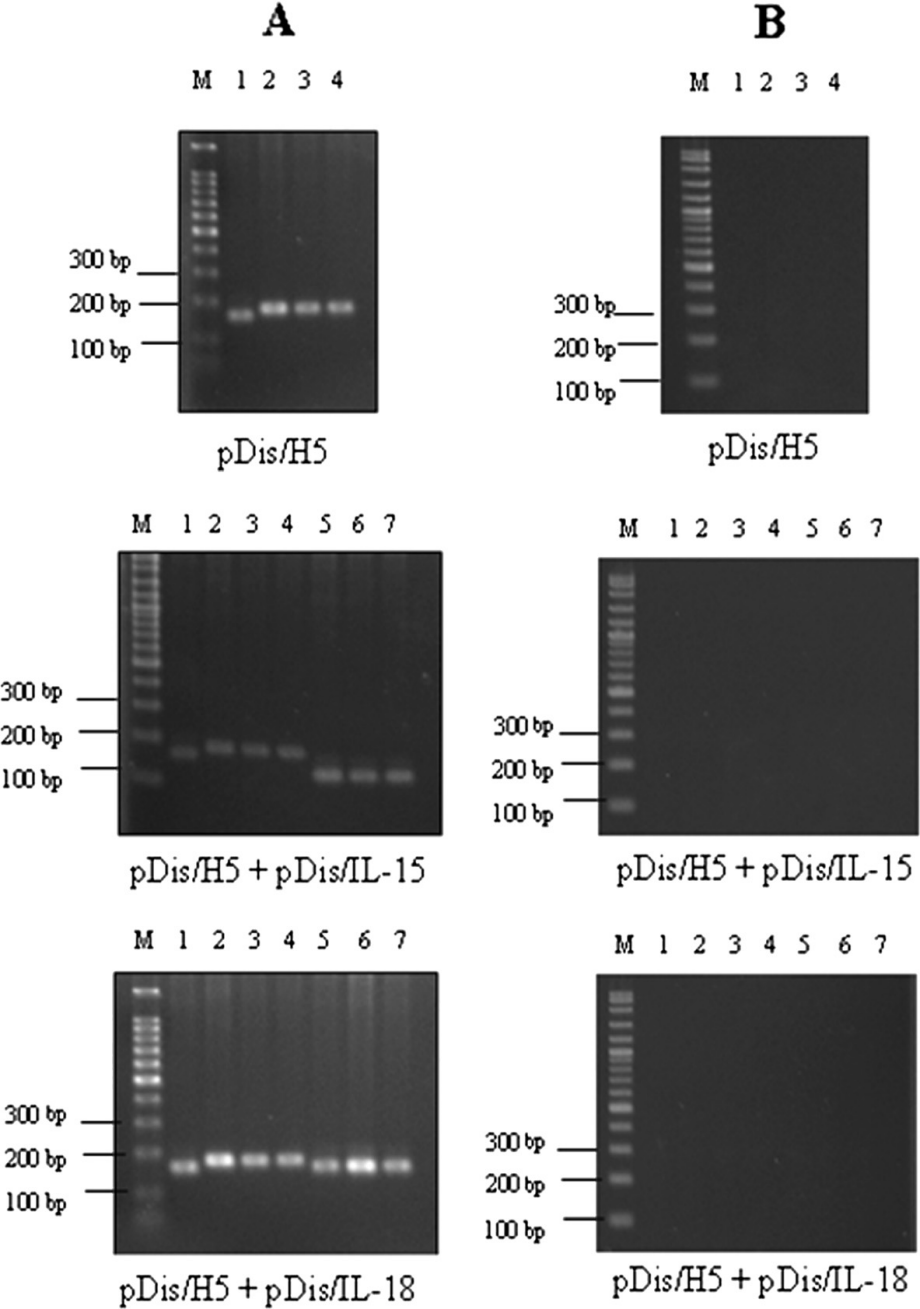
**RT-PCR analysis of mRNA extracted from spleen and muscles from chickens vaccinated with pDis/H5, pDis/H5 + pDis/IL-15 and pDis/H5 + pDis/IL-18.** The products were resolved in 1% (w/v) agarose gel in TAE. M: 100 bp DNA ladder (Promega, Madison WI) or VC 100 bp Plus DNA ladder (Vivantis, Malaysia) Lane 1: ACTB; Lane 2: H5 transcript in left muscle tissues; Lane 3: H5 transcript in right muscle tissues; Lane 4: H5 transcript in spleen. Lane 5: IL-15 or IL-18 transcript in left muscle tissues; Lane 6: IL-15 or IL-18 transcript in right muscle tissues; Lane 7: IL-15 or IL-18 transcript in spleen. In (**A**): RT-PCR analysis of mRNA. In (**B**): No specific band was detected when RNA from (A) was analyzed by PCR without the RT part.

The stability of the plasmid construct and the duration of the transgene expression in the immunized SPF chickens were determined by extracting the total genomic DNA and total RNA from chicken breast muscle and spleen for the PCR and RT-PCR analyses. The H5, IL-15 and IL-18 genes could be amplified by PCR and RT-PCR in groups immunized with the DNA vaccine, while chickens vaccinated with the empty plasmid, pDisplay, showed negative results. The PCR analysis on the extracted genomic DNA showed that the plasmid constructs were stable in the injected muscle as long as 6 weeks after vaccination. The transgene expression was reached systematically in the spleen and not only in the muscle at the injection site in both trials (Figure 6).

### GeXP analysis of gene expression in chicken spleens

A GeXP assay was performed to detect the expressions of 13 chicken cytokines including Th1, Th2 and pro-inflammatory cytokine genes (Table 3) in the spleen at the end of the experiment (56-day-old) after two DNA inoculations. A housekeeping gene, COL6A, was used to normalise the expression of the genes. The GeXP analysis showed that the expressions of TGF-β, GMCSF, IL-4, TNFSF13B, IL-1β, IL-15, IL-18, IFN-γ, IL-12β were detected in both trials. Except for IL-15 and IL-18, those genes showed a low level of gene expression ranging from 0.01 to 0.17 following the H5 DNA vaccination (data not shown). As shown in Table 8, the expressions of IL-15 and IL-18 in the control un-inoculated chickens were between 0.10 to 0.62. The level of the IL-15 gene expression in the pDis/H5, pDis/H5 + pDis/IL-15 and pDis/H5 + pDis/IL-18 groups in both trials (Table 8) were significantly higher compared to the control group (P < 0.05), except for the pDis/H5 + pDis/IL-18, which was not significant compared to the control group (P > 0.05) in the first trial. In addition, the pDis/H5 + pDis/IL-15 inoculated group showed the highest IL-15 gene expression in both trials compared to the other inoculated groups (P < 0.05). This showed that the co-administration of SPF chickens with pDis/IL-15 was able to induce a higher IL-15 gene expression than the pDis/H5 group (P < 0.05). Similar results were obtained for the IL-18 expression, where the pDis/H5 + pDis/IL-18 groups in both trials (Table 8) were significantly higher compared to the control group (P < 0.05). Meanwhile, pDis/H5, pDis/H5 + pDis/IL-15 did not induce a significant level of IL-18 compared to the control group (P > 0.05).

## Discussion

The *in vivo* studies using SPF chickens were conducted after confirming that all the plasmid constructs were able to express the desired proteins and were transcriptionally active in the *in vitro* expression analysis. In addition, the transgenes were stable over a period of 6 weeks after the booster dose, thus implying the potential that the plasmid DNAs encoding the target proteins could be expressed over a sustainable period.

Chickens inoculated with pDis/H5 + pDis/IL-15 at 4 weeks post inoculation in Trial 1 (Table 4) and Trial 2 (Table 5) showed a 2.82 fold and 4.87 fold increase in the HI antibody titer respectively, compared to pDis/H5 expressing the H5 alone (P < 0.05), suggesting that IL-15 plays a role in enhancing the humoral immune response. This finding was comparable with several studies conducted in different animal models where the recombinant vaccinia virus vaccine with integrated IL-15 expressing gp160 of human immunodeficiency virus generated a more robust and durable antibody response compared to gp160 alone [17]. In another study, the recombinant vaccinia virus-based pentavalent H5N1 AI vaccine adjuvanted with the human IL-15 induced stronger neutralizing antibodies against the AIV H5 in mice [18]. Another study demonstrated that the recombinant protein from the *Eimeria acervulina* in combination with the chicken IL-15 against coccidiosis which exhibited significantly greater serum IgG antibody levels than with the recombinant protein alone *in ovo*[19].

This study also showed an increased in the HI titer by the pDis/H5 + pDis/IL-18 at 4 weeks post inoculation in Trial 1 (Table 4) and Trial 2 (Table 5) by 2.14 fold and 2.00 fold respectively, compared to the pDis/H5 expressing the H5 alone (P < 0.05). Several reports have shown that the plasmid encoding IL-18, which is used as an adjuvant in mammalian vaccines, promote high antibody titers [20,21]. The recombinant chicken IL-18 has been shown to significantly enhance antibody responses to the *Clostridium perfringens* α-toxoid and NDV antigens [22]. Higher HI titers induced in chickens immunized with the Newcastle disease vaccine co-administrated with chicken IL-18 further confirm the immunostimulatory activities of chicken IL-18 *in vivo*[23].

The highest HI GMT of 16 ± 0.00 was observed in SPF chickens at 4 weeks post inoculation after two pDis/H5 + pDis/IL-15 inoculations were given in Trial 2. This is comparable to the studies conducted by our group where chickens immunized intramuscularly with the H5 DNA vaccine with genetic adjuvants, such as MDP-1, Esat-6 and HSP70 of the *Mycobacterium tuberculosis,* developed a higher HI titer compared to the H5 DNA vaccine alone [24-26]. However, all the previous studies required three vaccinations to achieve a comparable HI titer. Generally, the HI titer obtained in the present study was low compared to the following studies using inactivated AI vaccine. Chickens vaccinated with inactivated AI vaccine with HI GMT of 10 to 40 were associated with the prevention of mortality and HI GMT of 80 or more were associated with protection for mortality [27]. However, a previous study has shown that although the HA gene DNA vaccine induced a low HI antibody level after two DNA injections, immunized chickens were still protected against the lethal HPAI virus challenge [28], suggesting the role of other immune components, particularly in cellular immunity. Previous studies have shown that although the antibody against HA played a role in neutralizing viral infectivity, cellular immunity plays an important role in viral clearance [29,30].

The flow cytometry results for both trials (Table 6 and Table 7) demonstrated that pDis/H5, pDis/H5 + pDis/IL-15 and pDis/H5 + pDis/IL-18 were able to trigger higher CD4+ T cell production upon inoculation. The co-administration of SPF chickens with the pDis/IL-15 adjuvant was able to induce a higher production of CD4+ T cells than the pDis/H5 group (P < 0.05). These findings were consistent with previous reports that IL-15 has profound effects on the proliferation and survival of CD4+ T cells [31]. On the other hand, the present study revealed that pDis/IL-15 was not significant (P > 0.05) in inducing CD8+ T cells (Table 6). Nevertheless, in mammalian studies, IL-15 has been shown to support the growth of CD8+ T cells [32,33]. As shown in Figure 6 and Table 8, the presence of the IL-15 transcript in the spleen and muscles of chickens indicated that chicken IL-15 was able to express *in vivo* and the low level of CD8+ T cells was not due to the absence or low expression of the IL-15 protein.

The flow cytometry results in Trial 2 (Table 7) demonstrated that the co-administration of pDis/H5 + pDis/IL-18 was able to trigger a higher CD4+ T cell proliferation than the pDis/H5 group (P < 0.05). These findings were consistent with previous reports that IL-18 yielded stronger CD4+ T cell responses in immunized mice [13] and chickens [34] thus enabling DNA vaccines to achieve enhanced immune responses. On the other hand, the reports also showed that the co-administration of the IL-18 plasmid as an encoded adjuvant yielded stronger CD8+ T cells [35]. However, both trials in the present study revealed that pDis/IL-18 was not significant (P > 0.05) in inducing CD8+ T cells in the inoculation group. This indicated that IL-18 did not influence CD8^+^ T cell proliferation significantly, at least not in the PBMC samples at 2 weeks and 5 weeks post inoculation in Trial 1 and 3 weeks and 6 weeks post inoculation in Trial 2.

This study also suggests that the age of the SPF chickens at the time of inoculation could have influenced the immune response of the chickens, where chickens inoculated when they were 14 days old showed a higher HI antibody titer compared to those vaccinated when they were a day old (Table 4 and 5). This finding is comparable to studies which have shown that the priming of the SPF chickens at different ages played a significant role in determining the immune responses [36,37]. The results in this study showed that the transgenes were stable over a period of 6 weeks after the booster dose, thereby implying the potential that the plasmid DNAs encoding the target proteins could be expressed over a sustainable period. A previous study has shown that chickens vaccinated with the DNA vaccine resulted in the distribution of the target genes in the heart, lungs, liver, spleen and kidneys [38].

The cytokine expression levels of the immunized chickens were evaluated using GeXP analysis. The GeXP assay was designed originally for a high throughput and differential assessment of a multiplexed expression profile by a single RT-PCR [39]. The assay has a high dynamic linear range and improved sensitivity and specificity compared to other platforms [40]. The co-administration of the H5 DNA vaccinated SPF chickens with the pDis/IL-15 adjuvant was able to induce higher IL-15 gene levels than the control group (P < 0.05). However, only low levels of the IL-18 gene were observed among the treatment groups compared to the control group. Other cytokine genes including the TGF-β, GMCSF, IL-4, TNFSF13B, IL-1β, IFN-γ, IL-12β showed low levels of gene expression following the H5 DNA vaccination, probably due to the fact that the 56-day-old chicken spleens were collected at the end of the experiment.

In this study, we evaluated the immuno-regulatory effects of the co-administration of pDis/H5 + pDis/IL-15 or pDis/IL-18 via assessed on the peripheral blood CD4 and CD8 population and the cytokine expression levels. These results provide evidence that the pDis/IL-15 adjuvant was able to increase the CD4+ T cell population and stimulate the expression of IL-15 which might play a part in immunity against influenza via the induction and maintenance of CD8+ T cell memory, and providing help to B cells for antibody production. However, studies on the biological activities of IL-15 and IL-18 using the *in vitro* recall assay or evaluation on the regulation of cytokines on the NK cell (CD3-/CD8+) activity and interferon-γ production should be further appraised in the future together with the challenge of the H5N1 virus against the vaccinated chickens. These studies can contribute to the understanding on the role of the pDis/IL-15 adjuvant as a protection against the AIV.

## Conclusions

In conclusion, the results in this study demonstrate that pDis/H5 + pDis/IL-15 has the potential to be used as a DNA vaccine against AIV in chickens. Further studies are currently underway in exploring the possibility of conferring chickens immunized with pDis/H5 + pDis/IL-15 with protection against the challenge of the H5N1.

## Abbreviations

AIV: Avian Influenza Virus; FSC: Forward Scatter; FITC: Fluorescein Isothiocyanate; GMT: Geometric Mean Titer; HPAI: Highly Pathogenic Avian Influenza; IFAT: Indirect Immunofluoresence Antibody Test; IL: Interleukin; PBMC: Peripheral Blood Mononuclear Cells; SSC: Side Scatter; SDS: Sodium Dodecyl Sulphate; SPF: Specific Pathogen Free.

## Competing interests

The authors declare that they have no competing interests.

## Authors' contributions

ARO and KLL organized and designed the study; KLL performed the assays, analyzed the data, and wrote the manuscript; SDJ provided the primers for the quantification and detection of cytokines; SKY participated in the data analysis; NBMA, MHB, AI, ARO contributed to the technical aspects of the manuscript. All authors read and approved the final manuscript.
